# Forum Marketing in International Commercial Courts?

**DOI:** 10.1093/ojls/gqae022

**Published:** 2024-07-04

**Authors:** Georgia Antonopoulou

**Keywords:** international commercial courts, forum selling, forum marketing, pro-business approach, procedural inequality

## Abstract

Forum selling is a legal term used to describe the practices of courts and judges, geared towards attracting cases, such as increasing the predictability of judgments or speeding up trials. However, do courts also go beyond forum selling to attract cases? Taking international commercial courts as its focus, this article explores how these courts market themselves to attract cases and coins the term ‘forum marketing’. It demonstrates that the courts’ recent establishment, coupled with their voluntary jurisdiction, creates a compelling context, which encourages them to engage in forum marketing. The article argues that forum marketing is not merely a byproduct of the competition in commercial dispute resolution, but a powerful mechanism with deeply persuasive, normative and, effectively, structuring properties. Forum marketing is central to disseminating and reinforcing a pro-business approach in civil justice, consequently setting the stage for procedural inequality and a one per cent procedure.

## 1. Introduction

When laws allow litigants to ‘shop around’ for the most favourable forum to resolve their disputes, some courts may have an interest in attracting those disputes. It is argued that the prospects of benefits for the local economy, increased court revenue or prestige in a specific field of law could encourage certain courts and judges to make an active attempt to attract cases, thereby engaging in what has been referred to as ‘forum selling’.^1^ Courts practising forum selling enhance the quality and predictability of their judgments or expedite trials, ultimately with the aim of attracting a greater number of cases.^2^ While forum selling could prompt courts and their jurisdictions to compete with each other, consequently improving their court systems and laws, it is nevertheless argued that in the context of non-contractual disputes, forum selling could result in pro-claimant approaches. Since the claimants in non-contractual disputes choose the court and are the actual forum shoppers, forum selling courts grant claimants procedural advantages that could unfairly tilt the scales of justice in their favour.^3^

However, the practices of a new ‘species’ of courts, called international commercial courts, challenge the traditional accounts of forum selling and illustrate that some courts go beyond forum selling to attract cases. For instance, most international commercial courts present themselves as self-standing courts, even though they are merely chambers or divisions within existing courts. In addition, some of these courts have appointed reputable foreign judges to their bench, mostly drawn from England and Wales, and thereby seek the endorsement of internationally renowned legal figures, while others closely collaborate with the local bar. Such practices—the use of brand names, celebrity endorsement and public relations—exceed the definitional boundaries of forum selling and underline the need for a concept that better captures the practices of international commercial courts and their judges on the ground.

The aim of this article is to take the first step towards addressing this need. The article develops a novel concept and coins the term ‘forum marketing’. It illustrates that international commercial courts do not simply engage in forum selling, but openly promote their procedural advantages and reach out to prospective litigants through forum marketing. While forum selling and forum marketing are interrelated processes with the common aim of attracting cases, forum marketing exceeds the confines of the courtroom and reaches out to a broader audience of prospective litigants. Drawing from the numerous communication materials of international commercial courts, policy documents, legislation and interviews, this article argues that forum marketing is central to the functioning of international commercial courts. As recently established institutions, with their jurisdiction primarily based on choice-of-court agreements, international commercial courts engage in forum marketing to raise awareness of their recent establishment and cultivate a positive reputation in an increasingly competitive international dispute resolution market.

The study of forum marketing does not solely align academic theory with judicial practice. Shifting the theoretical lens from forum selling to forum marketing sheds a brighter light on the implications of international commercial courts as novel institutions. Forum marketing signals a change, revealing that the days when courts spoke through their judgments are fading. Forum marketing, in particular, reconfigures the principle of ‘open justice’ by introducing the idea that public courts and judges must become more visible and attractive to litigants. However, under certain circumstances, given that marketing tends to prioritise style over substance, forum marketing can also have the effect of deceiving and misleading litigants in their choice of court.

More significantly, this article argues that forum marketing is not merely a byproduct of the increasing competition in commercial dispute resolution, but a powerful mechanism with deeply persuasive, normative and, effectively, structuring properties. It is central to disseminating and reinforcing a pro-business approach to civil procedure and private international law, which could eventually create procedural inequality and tilt civil justice as a whole in favour of corporations and business litigants. Forum marketing achieves this in three ways. First, it encourages states and their courts to adopt procedural features modelled on common law courts. In this way, forum marketing sustains stereotypes of ‘good’ and ‘bad’ procedure, and is deeply implicated in the inequalities between Western and non-Western legal systems. Second, by throwing the spotlight on the procedural standards of a more market-oriented outlook, forum marketing empowers those litigants who can utilise the market and diverts our gaze from alternative procedural standards based on broader social values. Lastly, by presenting international commercial courts’ procedural features as a direct response to parties’ preferences, forum marketing obscures the politics behind the creation of international commercial courts and reinforces the misplaced assumption that civil procedure and private international law are technical fields of law that lack redistributive implications. Yet, this emphasis of forum marketing on a universalising mode of thinking, the primacy of market values and the separation of law and politics testifies to a neoliberal ideology in civil justice with broad redistributive implications. By connecting marketing studies to the study of international commercial courts, this article connects two seemingly disparate areas of knowledge and offers an innovative look at the practices of courts and their judges, which steers the study of international commercial courts into a new direction. In this way, the article contributes to the broader scholarly debates surrounding the role of the neoliberalised state and its legal institutions as a ‘market maker’.

The remainder of this article is structured as follows. Section 2 defines the concept of forum marketing and explains how it complements forum selling. Section 3 explains the methodology and, in particular, how the interviewees were selected and the interviews conducted. Section 4 then examines the reasons why international commercial courts engage in forum marketing, and section 5 subsequently lays down the various forum marketing practices of international commercial courts. Section 6 explores the implications of forum marketing for civil procedure and private international law. The last section concludes that forum marketing reinforces a pro-business approach in civil justice that enhances the influence of corporations and business litigants over civil justice, consequently setting the stage for procedural inequality and a one per cent procedure.

## 2. Conceptualising Forum Marketing

In recent years, various international commercial courts have been established in Europe and Asia.^4^ In Europe, these courts comprise the Netherlands Commercial Court (NCC), the Paris International Chambers and the Chambers for International Commercial Disputes, created in Frankfurt, Hamburg, Stuttgart and Mannheim. In Asia, the Dubai International Financial Centre (DIFC) Courts, the Singapore International Commercial Court (SICC), the Qatar International Court (QIC), the Abu Dhabi Global Market (ADGM) Courts, the Astana International Financial Centre (AIFC) Court and the China International Commercial Courts (CICC) are to be found. International commercial courts are national courts that specialise in cross-border commercial disputes. Being largely modelled on the London Commercial Court (LCC) and international commercial arbitration, they have new and innovative features, geared towards the particular needs of international commercial disputes. For instance, most international commercial courts use English as the language of their proceedings or have foreign judges—referred to as international judges—sitting on their bench.

The academic literature on international commercial courts highlights that, unlike ordinary courts, international commercial courts are the product of competition between civil justice systems. For example, in Europe, international commercial courts were established in the wake of Brexit with the aim of attracting commercial litigation away from London.^5^ Bookman notes that the competition between international commercial courts encourages them to attempt to attract cases, which can lead to forum selling.^6^ As mentioned previously, forum selling is a legal term used to describe the active attempts of some courts and their judges to attract cases.^7^ Courts and judges who have an interest in increasing their caseloads make themselves more attractive to litigants by adopting various forum selling techniques, such as raising the predictability of their judgments, speeding up trials or favouring the claimants who choose the court.^8^ According to the literature on international commercial courts, these courts engage in forum selling, mainly by relaxing jurisdictional requirements to facilitate the establishment of their jurisdiction, and by emulating international commercial arbitration to appeal to parties with a preference for commercial arbitration.^9^

However, the existing literature, while insightful, fails to fully account for the practices of international commercial courts. A closer look at these courts and their judges will reveal that some of their practices, such as branding and celebrity endorsement, do not fit squarely into the definition of forum selling. Instead, these practices are more concerned with how international commercial courts present themselves and are geared towards a broader audience of prospective litigants. Thus, it would appear that in the context of international commercial courts something beyond selling is at work.

As explained by Baker, a notable marketing scholar, ‘there are as many definitions of marketing as there are people willing to make one’.^10^ In fact, the boundaries of marketing are fuzzy, due to its overlap with other company activities and evolution over time. Armstrong and Kotler define marketing as the process through which companies engage customers, build strong customer relationships and create customer value, in order to capture value from customers in return.^11^ Selling represents part of that process, but it is only ‘the tip of the marketing iceberg’.^12^ Thus, marketing complements selling by retaining customers, while at the same time expanding sales by raising awareness and reaching out to new customers.^13^

Companies utilise a variety of tools in their marketing strategies, commonly referred to as the ‘four Ps’: product, price, place and promotion.^14^ The company must first create a *product* that satisfies customers’ needs. It must then *price* that product and decide how to *place* it in the relevant market. Finally, it must *promote* the product by communicating it to potential customers and persuading them of its merits. In 1969, Kotler and Levy’s seminal article ‘Broadening the Concept of Marketing’ extended the marketing concept to non-business entities, arguing that marketing principles are transferable to not-for-profit organisations, political parties and even ideas, provided that exchange and relationship activities are involved.^15^ In this way, the above authors illustrated marketing as a pervasive societal activity that ‘goes considerably beyond the sale of toothpaste, soap, and steel’.^16^ In light of globalisation, marketing is further growing in significance because it allows companies to connect with customers, irrespective of the geographic distance. It thereby serves as a means of participating effectively in the global competition for products and services.^17^

In a similar vein, while forum marketing and forum selling share the aim of attracting cases, forum marketing is broader than forum selling. Forum marketing exceeds the confines of the courtroom and reaches out to a broader audience of prospective litigants. This article mainly focuses on the promotional aspects of international commercial courts’ forum marketing, corresponding to the fourth of the four Ps of marketing strategy. In line with marketing studies, international commercial courts engage in marketing to raise awareness of their existence as recently established institutions and to cultivate a positive reputation among potential litigants, who may then choose in their favour. Since international commercial courts specialise in cross-border disputes and aim to attract parties from different parts of the world, forum marketing facilitates their access to an international audience and enables them to participate in the global competition for courts and judicial services.

While international commercial courts have attracted the attention of legal scholars, an analysis of their marketing is missing. This could be owed to a more traditional account, according to which civil courts are anonymous institutions, disinterested in public appearances. To date, the academic literature on the marketing of dispute resolution methods is limited and has mainly focused on the marketing practices of international commercial arbitration^18^ or the International Criminal Court.^19^ However, unlike arbitral tribunals, international commercial courts are public courts and are therefore—albeit only in principle—immune to the competitive market pressures that are inherent in arbitration. Unlike the International Criminal Court, international commercial courts are national courts. As such, international commercial courts’ institutional mould and normative purpose are very different from those of international commercial arbitration or international criminal tribunals. Thus, studying the marketing of international commercial courts permits an exploration of marketing practices in a previously unexamined setting. Admittedly, one could point out that some national courts, notably, national supreme or constitutional courts, engage in judicial communications. National courts engage in judicial communications to inform the public of recent case law and legal developments, and to promote the public’s understanding of issues of legal and constitutional importance.^20^ By contrast, international commercial courts are lower courts of first instance, with judgments that primarily focus on the more mundane aspects of private law disputes, where broader public interests or the morality of crime and punishment are rarely, if ever, relevant. In contrast to judicial communications, forum marketing has the distinct aim of raising awareness and cultivating a positive reputation, ultimately with a view to attracting cases. With more and more countries contemplating the establishment of international commercial courts and being willing to participate in the global competition of courts and civil justice systems,^21^ international commercial courts offer a compelling context in which to examine the marketing of courts and its implications.

## 3. Methodology

To explore forum marketing in the context of international commercial courts, I first investigate the courts’ communication materials, such as official webpages, social media accounts, press releases and newsletters. I then turn to the various policy documents that preceded the establishment of international commercial courts, the legislation and court rules, as well as the academic literature that followed their creation.

In order to facilitate understanding of how the forum marketing of international commercial courts works, the article is also indirectly informed by semi-structured interviews conducted with judges sitting at an international commercial court, court personnel and lawyers that have appeared before an international commercial court. Since international commercial courts are novel institutions, interviews were arguably the most suitable way to explore and better understand their establishment and the nature of their practices.

Unlike the rest of the analysis, the interviews focus on two courts: the NCC and the SICC. The NCC was chosen because it has more innovative features than other international commercial courts in Europe. For example, it uses English as the court language throughout the procedure, including pronouncement of the judgment. In addition, a glimpse at the court’s website indicates that, also unlike other international commercial courts in Europe, the NCC engages in forum marketing by regularly releasing newsletters, maintaining a social media account and even featuring a promo video on its website. In a similar vein, the SICC was chosen as the NCC’s Asian counterpart. Unlike other international commercial courts in Asia, it is a court with multiple innovative features that actively promotes itself. Furthermore, the SICC is located in an entrepreneurial jurisdiction that openly states and pursues its aim of rising to become an Asian dispute resolution hub.

Seventeen interviews were conducted in total from May 2019 to September 2020. The interviewees were identified from the courts’ websites and case law. In some instances, I directly contacted the interviewees by email, personally approached them in court or was introduced to them by previous interviewees and existing contacts. The majority (12) of the interviewees were lawyers, while the rest were judges at these courts and court registrars. Eleven interviews were conducted in person in the Netherlands and Singapore. Owing to the outbreak of the COVID-19 pandemic, the remaining interviews were conducted online, via Zoom or Skype. Apart from two interviews in which the interviewees did not feel comfortable being recorded, all interviews were recorded and transcribed. In addition, notes were taken.

At the start of the research, the interviews mainly focused on the functioning of the courts and the legal practitioners’ perception of the courts’ operations. However, as the topic of marketing surfaced in the process of interviewing, I adjusted the questions to address this subject. Given that the marketing of civil courts is a novel phenomenon, the interview questions were exploratory and mainly focused on why and how exactly international commercial courts engage in marketing. It should be noted that some interviewees were reluctant to answer questions relating to forum marketing. This reluctance makes sense if we consider that not only is the traditional perception of courts, such as public institutions, immune to any market(-ing) pressure, but also that forum marketing is an ‘uncomfortable’ topic that may harm the reputation of these nascent courts by portraying them as ‘attention seeking’ courts.

## 4. The Motives for Forum Marketing

While different reasons drove the establishment of international commercial courts around the world, the aims of attracting foreign investment and generating litigation business were fundamental forces in their creation. The economic reasons behind the establishment of international commercial courts encourage the courts to make an active attempt to attract cases and at the same time translate into forum marketing motives. Furthermore, some motives for forum marketing are related to international commercial courts’ institutional and procedural features. The fact that international commercial courts are recently established institutions lacking compulsory jurisdiction motivates them to engage in forum marketing in order to raise awareness of their establishment, cultivate a positive reputation, distinguish themselves from other dispute resolution methods and ultimate persuade parties to rewrite their dispute resolution clauses in their favour.

### A. Benefits for the National Economy

The academic literature identifies various motives as to why some courts have an interest in attracting cases and engaging in forum selling. The main motives for forum selling are benefits for the local economy and the prospects of increased court revenue.^22^ International commercial courts indeed benefit a country’s economy by improving the business climate and creating business for the legal services sector, and this therefore poses a powerful incentive to attract cases and engage in forum marketing.

The World Bank’s Doing Business reports exemplify how specialised commercial courts benefit national economies. According to the reports, commercial courts facilitate contract enforcement and are therefore regarded as being conducive to a good business climate.^23^ One of the explicit aims of some countries that established international commercial courts was, indeed, to climb the ladder of the World Bank rankings.^24^ The rationale of improving the business climate and thereby attracting foreign investment is especially evident in the case of international commercial courts in the Middle East and Kazakhstan. These courts are established in Special Economic Zones and are part of broader strategies to attract offshore corporate and financial services.^25^ As Lord Woolf, former Chief Justice of the AIFC Court and first president of the QIC, explained, the courts are designed to build confidence in investors and appeal to capital markets.^26^ Similarly, the fact that international commercial courts in Europe were established in the wake of Brexit indicates that the courts could be one of the many ‘attractions’ to lure corporations away from London.

Except for benefiting and attracting foreign investors, international commercial courts are also expected to benefit domestic companies. The NCC’s establishment, for example, was also based on the expectation that it would save Dutch companies, especially small and medium-sized enterprises, the costs of litigating abroad, especially before the more expensive English courts, or going through international commercial arbitration.^27^

Investment attraction considerations aside, international commercial courts may directly benefit national economies. They attract litigation and thereby business for the legal services sector. Legal services contribute directly to GDP as a result of law firms’ turnover and third-party funders. For instance, the SICC was established as part of Singapore’s broader policy objective to become Asia’s dispute resolution hub.^28^ In a conference speech in 2015, the former Senior Minister of State for Law underlined that the SICC aimed to attract disputes and thereby boost legal services.^29^ Finally, it is expected that attracting litigation will translate into business for hotels, restaurants, couriers and translation services.^30^

Thus, the prospects of benefits for national economies, particularly the considerations of attracting investment and creating business for legal services, encourage international commercial courts to engage in forum marketing in order to attract cases and present the first forum marketing motive.

### B. Increased Court Revenue

Courts usually charge fees which only cover costs and leave little room for profit. Legal scholars therefore claim that while the prospects of increased court revenue could explain why some courts actively attempt to attract cases, other motives, such as the prospects of increased reputation, offer a better explanation for forum selling.^31^ However, some international commercial courts impose higher fees compared to ordinary courts within the same jurisdiction. This means that international commercial courts, unlike ordinary courts, have a powerful profit-making motive to attract cases and engage in forum marketing.

In 2022, the fees for the NCC were €15,856 per party, and €21,141 per party on appeal.^32^ The NCC thus charges court fees that are significantly higher than the court fees charged by the rest of the ordinary Dutch courts.^33^ Similarly, the SICC levies higher court fees calculated on the basis of a distinct fee schedule.^34^ The latest legislative proposal for the establishment of specialised chambers in Germany slightly raises fees for high-value claims.^35^

The justification for higher court fees has to do with establishing international commercial courts in a budget-neutral way, where parties themselves bear the costs of high-value, complex and time-consuming litigation. However, the fees of international commercial courts not only cover costs, but may also result in profit. A market survey conducted by the Boston Consulting Group before the NCC was established estimated that in the long term the NCC should annually receive 100 cases on first instance and 25 cases on appeal.^36^ This means that by the time the NCC indeed reaches this approximation, the start-up costs, estimated at €3.8 million, will be repaid and the NCC will start making a profit. According to the parliamentary discussions that preceded the court’s establishment, the NCC revenues would be used to cross-subsidise the ordinary Dutch courts.^37^ It therefore becomes apparent that some international commercial courts could generate revenue by imposing increased court fees and therefore possess the economic motive to engage in forum marketing.

### C. Increased Judicial Reputation and Income

As regards judges, although a greater caseload does not increase judicial salaries, legal scholars claim that other non-monetary motives encourage judges to compete for cases. These motives are mainly the intellectual challenge of handling complex cases and an increased reputation.^38^ However, the literature contends that one cannot completely rule out the prospects of indirect economic gain. Judges may derive additional income from ‘off the bench’ activities, such as authoring books or giving speeches.^39^

As in the case of forum selling, the intellectual challenge of handling complex cases and the prospects of an increased reputation are additional reasons that international commercial courts’ judges engage in forum marketing. Being appointed as a judge to an international commercial court signals expertise in international commercial dispute resolution and elevated standing in the judicial community. A glance at the international judges appointed to these courts indeed reveals that they are internationally renowned legal figures.^40^ Although international commercial courts in Europe are staffed by national judges, the fact that these were appointed to an international commercial court because of their increased expertise and English language skills singles them out among their peers and boosts their reputation.^41^

However, in the context of international commercial courts, international judges’ case-dependent remuneration offers an additional explanation for their forum marketing endeavours. International commercial courts in the Middle East, Kazakhstan and Singapore have appointed foreign nationality judges to their bench. A different appointment and remuneration regime applies to international judges. International judges lack tenure and are appointed for a fixed period on the basis of private contracts. According to informal discussions, their remuneration is calculated based on an hourly fee for the time spent on each case assigned.^42^

International judges’ case-dependent remuneration means that these judges profit from an increased caseload and may have an economic interest in being appointed to the bench of an international commercial court, assigned to cases and reappointed once their contract expires. In addition, some international judges are at the same time practising lawyers or arbitrators. Serving at an international commercial court that has successfully acquired a significant caseload may therefore translate into more legal business and appointments as arbitrators. Further, some international judges have transitioned from one international commercial court to another or are serving simultaneously at various international commercial courts. Side activities such as authoring or editing books on international commercial courts and speaking engagements may add to international judges’ income prospects.

Contrary to the literature on forum selling, according to which judges’ interest in an increased caseload mainly lies in reputational motives, international judges’ case-dependent remuneration and parallel activities suggest that these judges also have a strong economic interest in attracting cases and forum marketing.

### D. Forum Marketing Specific Explanations: Raising Awareness

Yet, there are some explanations for forum marketing that are intrinsic to the marketing of international commercial courts. In the context of international commercial courts, forum marketing is also explained by the fact that these courts are novel institutions and therefore need to raise awareness of their recent establishment. Forum marketing also finds its explanation in the fact that international commercial courts are extremely reputation-sensitive.

The SICC, the CICC, the Paris International Chambers, the German Chambers for International Commercial Disputes and the NCC lack compulsory jurisdiction and their jurisdiction depends on the parties’ choice.^43^ The voluntary jurisdiction of these courts limits the number of incoming cases and challenges the courts to acquire a caseload. Although the international commercial courts in the Middle East and Kazakhstan are vested with compulsory and exclusive jurisdiction over disputes linked to the respective zones, being chosen in a choice-of-court agreement is significant for these courts as well. It adds international disputes to their predominantly domestic caseload and therefore increases the number of incoming cases.

Moreover, the coexistence of various dispute resolution venues offers parties a wide range of forum shopping options. Parties in international commercial disputes may choose an international commercial court, stick to the ordinary courts or resort to arbitration. Yet, unlike ordinary courts and arbitration, international commercial courts are novel institutions, and as a result, parties may still be unaware of their existence as courts. The author asked a court member why they promote and market the international commercial court more than the rest of the national courts and they replied: ‘In the rest of the courts, cases do not come to you on a voluntary basis. Therefore, we have to make people aware of what we are doing.’^44^ In a similar vein, another court member underlined:

One party may say: let’s go to the [name of international commercial court]. And the other party might say: Oh, I never heard of it. We crossed the first hurdle, which is awareness. We crossed the second hurdle, which is the recommendation of our jurisdiction clause. So now we are starting to see results regarding the third hurdle, which is that both parties say: Ok, let’s agree on a [name of international commercial court] jurisdiction clause.^45^

International commercial dispute resolution and the various forum shopping options available to parties challenge international commercial courts, most of which were created recently. In order to acquire a significant number of cases, they need to: raise awareness as to their existence; stand out from other well-established dispute resolution methods; and, consequently, carve out their own piece of international commercial dispute resolution. In order to achieve this goal, international commercial courts are forced to rise above the competition by engaging in forum marketing.

### E. Forum Marketing Specific Explanations: International Commercial Courts as Reputation-Sensitive Courts

Furthermore, international commercial courts are aware that the reputation factor largely drives parties’ choice of court. Reputation becomes especially important in the international context, within which international commercial courts operate and where greater distance between the courts and the litigants exists. All this means that international commercial courts are extremely reputation-sensitive and offers the last explanation why they engage in forum marketing.

In particular, international commercial courts have adopted various innovative features so as to distinguish themselves from ordinary courts and ultimately to attract disputes. These features predominantly aim at enhancing the courts’ expertise in cross-border commercial disputes and increasing procedural flexibility. Nevertheless, international commercial courts are aware that parties’ court preferences are not entirely based on the procedural merits of the chosen court.^46^ Parties choose a court for a variety of reasons, such as the quality of the judges or the speed of the dispute resolution.^47^ However, some of these factors are unrelated to the quality of a court and the justice system surrounding it. After all, comparing courts and justice systems is a complex, laborious and time-consuming task, with an uncertain outcome.^48^ Familiarity with a court or established market practices equally motivate parties’ choice of court.^49^ It would therefore be misguided to think that the founders of international commercial courts gathered around a table, deliberated for hours, mastered the subtle variations of procedural laws and came up with the optimal institutional and procedural features of international commercial courts. They did not—just as lawyers do not when drafting dispute resolution clauses.^50^ Based on the assumption that parties tend to gravitate towards the familiar and are often guided by a court’s sheer reputation, international commercial courts and some of their features primarily aim at sending signals of quality^51^ and at evoking a sense of familiar procedures.^52^

The above reveals that the forum marketing motives of international commercial courts do not neatly map onto the forum selling motives identified in the academic literature. The fact that international commercial courts operate on the international plane amplifies the economic and reputational motives for forum marketing. In addition, the case-dependent remuneration of international judges adds an economic motive to their forum marketing practices. Lastly, some explanations for forum marketing are particular to international commercial courts. The fact that the courts are recently established institutions as well as reputation-sensitive encourages them to engage in forum marketing in order to raise awareness of their establishment and cultivate a positive reputation.

## 5. Forum Marketing Practices

International commercial courts engage in various forum marketing practices in order to attract cases. The courts use brand names, seek the endorsement of internationally renowned legal figures, associate themselves with the established reputation of the cities hosting them and collaborate closely with the bar. In addition, some of the courts’ innovative features, such as the use of English as the court language or the adoption of arbitration-inspired features, are limited in scope and added value. They therefore mainly contribute to the marketing of international commercial courts by sending signals of quality and familiarity to foreign litigants.

The forum marketing of international commercial courts is vested in the hands of their judges and court personnel. Due to their expertise and network, judges are frequently involved in public-facing activities, which include the delivery of presentations and speeches, receiving foreign delegations and authoring publications. In addition, registry personnel and executive teams assist the judges in these courts with forum marketing. For instance, alongside their regular tasks, NCC law clerks and registrars are additionally tasked with assisting judges with public relations.^53^ The DIFC Courts have a Communications Department;^54^ the SICC has a Senior Director and an Assistant Director for Business Development;^55^ and the AIFC Court has a Managing Director of Communications and Events.^56^ As one interviewee explained, a marketing agency with expertise in public branding was once hired to assist with the design and production of marketing material.^57^

### A. Brand Names and Secondary Branding Association

One of the marketing practices refers to branding, especially the use of brand names. Brands, in the form of a name, symbol or design, or some combination thereof, enable consumers to identify products and services, thereby eliminating the need for consumers to analyse and compare alternative products and services.^58^ In the case of international commercial courts, the use of brand names makes it easier for litigants to identify the courts and therefore creates recognition.^59^ Although this article uses the term ‘international commercial courts’ indistinctively, the European international commercial courts or the SICC are not self-standing courts but are actually chambers or divisions within existing courts. Presenting international commercial courts as self-standing courts raises their visibility and portrays them as having a distinct advantage over the rest of the chambers or divisions within a court.^60^

The organisational set-up as chambers within courts allowed the rapid set-up of international commercial courts, while avoiding the time and costs of establishing separate courts. For instance, the NCC calls itself a court, but it is actually a court chamber of the Amsterdam District Court.^61^ Similarly, the SICC is a division of the General Division of the Singapore High Court, which in turn is part of the Supreme Court of Singapore.^62^ Inspired by the LCC, the SICC is a division of the High Court but is branded as a separate commercial court. Yip observes that branding the court as a stand-alone court conveys a sense of detachment from the national judicial system and gives the impression of it being a separate and therefore novel court.^63^

Whether international commercial courts are self-standing courts or chambers within courts is not merely a matter of nomenclature—it has repercussions regarding their procedural rules. Being chambers within courts influences the jurisdiction of international commercial courts by hinging their jurisdiction on that of the court of which they are a part. The chamber’s international, territorial and subject matter jurisdiction presupposes the international, territorial and subject matter jurisdiction of the court. Moreover, while European international commercial courts additionally make their jurisdiction dependent on the parties’ agreement, their establishment as chambers calls into question whether such agreements are choice-of-court agreements or merely agreements in favour of a chamber within a court.^64^ The answer to this question is decisive for the applicability of international, European or national rules on choice-of-court agreements and is central with regard to the binding force of agreements in favour of these courts.^65^

In addition to the use of brand names, some international commercial courts attempt to increase their recognition by establishing links to the reputation of the cities in which they are located—a practice referred to in marketing studies as secondary brand association.^66^ The NCC is an example of an international commercial court that makes use of such a marketing practice. In particular, the official website of the NCC hosts a video with the court’s main features on display.^67^ According to the video, the court is situated in Amsterdam, ‘a city with a proud history of commerce and justice’. This link between the NCC and Amsterdam indicates that the marketing of the court is intertwined with the marketing of the city in which it is located. The reputation of the latter boosts recognition of the former. Nonetheless, the link between the marketing of international commercial courts and that of the cities hosting them makes sense when one considers the cities’ established reputation and the fact that commercial disputes with cross-border elements tend to cluster in bigger cities with economic and business activities.

In the case of the CICC, the cities hosting the courts hold in addition a symbolic value. The First China International Commercial Court is in Shenzhen, while the Second China International Commercial Court is in Xi’an. Both cities are included in the ‘Belt and Road Initiative’; Shenzhen is considered the starting point of the ‘Belt’ route, while Xi’an is the starting point of the ‘Road’ route. The symbolism of both cities is so strong that it found its way into the jurisdiction of the courts. Disputes with a territorial connection to the ‘Belt’ route fall under the jurisdiction of the Shenzhen court, while disputes with a connection to the ‘Road’ route fall under the jurisdiction of the Xi’an court.^68^ This unclear division of jurisdiction between the first and second Chinese international commercial courts might be of minor practical relevance, since parties may choose the court they prefer despite the lack of a territorial link;^69^ nevertheless, the vague demarcation of jurisdiction between the courts highlights that in their communications the Chinese courts prioritise symbolism over procedure.

### B. Celebrity Endorsement

In addition to using brand names and capitalising on the reputation of the cities hosting them, some international commercial courts market themselves by seeking the endorsement of internationally renowned legal figures.

The DIFC Courts, the QIC, the ADGM Courts, the AIFC Court and the SICC host international judges on their benches, mostly drawn from common law jurisdictions. Most of the international judges were formerly judges in jurisdictions such as Australia, Hong Kong or England and Wales, and, in particular, the LCC.^70^ It is claimed that the involvement of foreign judges enhances judicial expertise regarding international commercial matters and facilitates the application of foreign law.^71^ While the CICC is exclusively composed of Chinese judges, it nevertheless features an International Commercial Expert Committee. The committee members are Chinese and foreign legal experts, and take on the task of mediating disputes and providing advisory opinions on specialised legal issues.^72^

However, the international judges’ expertise in commercial or foreign law does not provide the full picture as regards their appointment. Some judges sitting on the international commercial courts or the Chinese International Commercial Expert Committee—even if primarily appointed for their expertise—contribute to the marketing of these courts owing to their international reputation and their extended network. In some instances, international commercial courts even share their human and symbolic capital by exchanging judges among themselves. In addition, some international commercial courts feature arbitration academics and practitioners and thereby hint at the strong ties international commercial courts aim to forge not only among each other, but also with the arbitration community.^73^

Judges at the international commercial courts, the ‘grand old men’,^74^ have endorsed the establishment of these courts by lending them their expertise and reputation. Especially in jurisdictions that are often dismissed as authoritarian or whose judicial system lacks international standing, international judges add the recognition and credibility of their established judicial systems and mitigate the relatively short history of international commercial courts in dispute resolution.

### C. Public Relations

Central to the establishment and the functioning of international commercial courts is also their public relations engagement, in particular their collaborative relationship with the bar. International commercial courts sought the input of lawyers, and their initial caseload was largely dependent on a domestic buy-in. Legal practitioners in some instances participated in drafting the rules of international commercial courts and provided their opinion on the courts’ institutional and procedural design.^75^ London-based law firms advised the Dubai government on the DIFC Courts.^76^ Singapore’s ‘Big Four’ law firms participated in the SICC Committee and made recommendations with regard to the court’s features.^77^ The central role of the local bar in the rule-making process is also evident in the example of the Paris International Chambers. The Chambers’ procedural rules can be found in the respective ‘Protocols’, which are agreements signed between the Paris Bar, the Commercial Court and the Court of Appeal.

With regard to their caseload, although the courts ultimately aim to attract disputes not necessarily related to their jurisdiction, during their first years of functioning it is anticipated that the majority of cases will derive from the ordinary courts. For instance, the Boston Consulting Group market survey emphasised with regard to the NCC that during the court’s start-up years the majority of cases would be those involving a Dutch and a foreign company currently submitted to the ordinary Dutch courts.^78^ It therefore becomes apparent that at least the initial caseload of international commercial courts largely relies on the support of the local bar. For that reason, it is key for international commercial courts’ caseload to cultivate and maintain a close relationship with legal practitioners through public relations.

At the same time, the marketing of international commercial courts facilitates the marketing for legal practitioners. While the newly established courts were looking forward to hearing their first disputes, some lawyers were looking forward to appearing first before an international commercial court. After appearing before the newly established courts, they boasted a new field of expertise—that of litigating before an international commercial court. One lawyer explained:

There was quite a lot of attention. For instance, one of the colleagues, as soon as the hearing began, took a picture and put it on LinkedIn and within one day it was liked, like 100 times. And in the weeks afterwards, every time I met lawyers from other firms they were: Oh, you were before the [name of international commercial court]. So yes, it is … it has some commercial value appearing before the [name of international commercial court].

Well, my firm does a lot of referral work. A lot of cases come into this firm from other lawyers when there is a conflict of interests or if they do not have specific knowledge of the case. Therefore, to be known as one of the lawyers who has appeared before the [name of international commercial court] might result in other work coming in. But it also depends … at some point it will either become quite rare for the [name of international commercial court] to have any proceedings and then it has not so much value or it will become quite common and then it does not really matter. So, it is just for now. Hopefully, if some foreign party will appear before the [name of international commercial court] they will think of the lawyers that have experience before it.^79^

However, the same lawyer also expressed some scepticism about bringing their case before an international commercial court. As they explained, they were worried that their case might become a ‘showcase’, a platform for the court to broadcast its innovative features.^80^

Thus, it becomes apparent that international commercial courts have developed a close and collaborative relationship with the legal profession. This facilitates the courts in acquiring a caseload and in some instances might result in puffery and marketing narratives that obscure the courts’ actual procedural merits.

### D. Procedure As a Signal

The last forum marketing practice is the adoption of procedural features which primarily aim at sending signals of quality and familiarity to prospective litigants. International commercial courts have adopted various innovative features, such as the use of English as the court language, the application of common law and arbitration rules and practices, and the signing of Memoranda of Guidance. However, in the case of some courts the actual scope and the added value of these features are limited. These features therefore mainly contribute to the marketing of international commercial courts by sending signals of quality and familiarity to foreign litigants.

#### (i) English as the court language, common law and arbitration features

One of the innovative features of international commercial courts is the use of English as the language of court proceedings. Except for the CICC, which conducts trials in Chinese,^81^ and the SICC, which is located in an English-speaking jurisdiction, the rest of the international commercial courts have introduced English as the court language. It is claimed that English should enter the court room in order to facilitate the resolution of cross-border commercial disputes by saving parties from translation costs, easing the communication among parties and their foreign parent companies, and rendering public court proceedings understandable and therefore more accessible to foreign parties.^82^

However, the limited scope of English as the court language before some international commercial courts and its limited attractiveness to foreign litigants hint at its primarily marketing value. In particular, while some international commercial courts allow for a full-fledged trial in English, others confine its use to specific parts of the procedure. The Frankfurt Chamber is among the international commercial courts that use English in a limited manner. The use of English is limited to the oral hearing and does not extend to procedural documents, such as the statements of claim and defense, the protocol or the court’s judgment.^83^ Similarly, the use of English before the Paris Chambers mainly refers to documentary evidence.^84^ Furthermore, the potential of English to draw litigants to public courts is limited. Previous attempts to introduce English in the courts of Rotterdam, Cologne, Aachen and Bonn found little success and highlighted the fact that language on its own bears limited potential to attract disputes.

The limited scope and attractiveness of English as the court language before these European international commercial courts shows that while English may assist the newly established courts to familiarise themselves with their international constituency, it does not suffice on its own to draw litigants to continental courts unless it is accompanied by other procedural traits.^85^ By adopting English as the court language, international commercial courts mainly aim at signalling to foreign litigants the quality and familiarity of their proceedings.

Similar to the use of English as the court language, although the European courts borrow certain rules and practices from common law courts and arbitration, these are scarce and limited in scope. This fact might be explained if we consider that the European courts were the product of limited legislative amendments and cautious spending. They apply national rules of civil procedure that leave little room for the application of common law or arbitration rules. The limited scope of their features reveals that they have a forum marketing value. Being aware that parties usually resort to common law courts or arbitration for the resolution of their international commercial disputes, the continental international commercial courts invoke common law or arbitration features, ultimately aiming at signalling the quality and familiarity of their procedures to foreign parties. In the case of some international commercial courts, their English style or arbitration characteristics are therefore merely ‘buzz’ words aimed at appealing to parties versed in English-style litigation and arbitration practices.^86^

#### (ii) Memoranda of guidance

Another feature of international commercial courts that contributes to their marketing are Memoranda of Guidance. The Memoranda of Guidance are court-to-court agreements aimed at providing general information concerning the enforcement of each party’s money judgments in the other party’s courts. In doing so, they mainly improve the parties’ confidence in the recognition and enforcement of international commercial courts’ judgments abroad.^87^

Indeed, one of the main challenges facing international commercial courts is the recognition and enforcement of their judgments abroad.^88^ While international commercial arbitration is often preferred by parties due to the nearly worldwide reach of the New York Convention,^89^ the absence of such a widely applicable treaty or convention for foreign court judgments makes the recognition and enforcement of international commercial courts’ judgments more challenging. Recent developments, such as the Hague Conventions on Choice of Court Agreements^90^ or the Hague Convention on the Recognition and Enforcement of Foreign Judgments in Civil and Commercial Matters,^91^ hold a promising potential, but for the moment their territorial scope is limited.

Being one of the first international commercial courts, the DIFC Courts were faced early on with concerns over the recognition and enforcement of their judgments.^92^ So, to enhance the recognition and enforcement of their judgments, the DIFC Courts have signed various Memoranda of Guidance with courts in foreign jurisdictions.^93^ Similar to the DIFC Courts, the SICC has signed Memoranda of Guidance on the enforcement of money judgments with commercial courts in other jurisdictions.^94^ When I asked whether the legally non-binding character of the memoranda could question their potential to improve the recognition and enforcement of SICC judgments, SICC court personnel replied, echoing similar statements made in the context of the DIFC Courts, that it is unlikely that courts will ignore a memorandum signed by the chief justices of the signing jurisdictions.^95^

Despite these claims, the legally non-binding character of the Memoranda of Guidance calls into question their effectiveness. It therefore remains to be tested whether the Memoranda of Guidance have the potential to improve the recognition and enforcement of court judgments and their usefulness apart from them being a marketing practice able to improve public perceptions on the receptivity of the international commercial courts’ judgments abroad.^96^

## 6. The Implications of Forum Marketing

The various practices that characterise forum marketing illustrate that all international commercial courts, whether in Europe or Asia, engage in this activity. As the lack of a significant number of incoming cases would otherwise call into question the need for these international commercial courts, forum marketing is central to their continued existence and future development. By encouraging international commercial courts and their judges to reach out to prospective litigants, forum marketing comes with implications that exceed the immediate context of the forum marketing phenomenon.

### A. Misleading Marketing

As noted above, some of the procedural advantages of international commercial courts are limited in scope. This applies to the use of English as the court language, and the common law and arbitration features of the European international commercial courts. Similarly, the potential of the Memoranda of Guidance to enhance the recognition and enforcement of international commercial court judgments abroad is limited, since the memoranda lack a legally binding effect.

Cuniberti points out that Memoranda of Guidance may even mislead parties in choosing an international commercial court.^97^ Since the recognisability and enforceability of court judgments abroad is a significant factor in choice of court and dispute resolution methods, parties could choose a particular international commercial court in the erroneous belief that its judgments will be recognised and enforced abroad on the basis of a Memorandum of Guidance. Cuniberti therefore notes that misrepresenting the enforceability of international commercial court judgments could be characterised as misleading or deceptive advertising. Deceptive advertising normally results in public enforcement or tortious liability remedies. However, given that these remedies are designed to regulate the practices of private actors, they are inapplicable in the case of international commercial courts.^98^

What is illustrated by the above is that forum marketing can mislead parties in their choice of court by overstating the procedural merits of courts and functioning as misleading or deceptive marketing. International commercial courts could mislead parties by giving them the impression that English is used throughout court proceedings, that they incorporate multiple features from common law courts or international commercial arbitration and that the Memoranda of Guidance are sufficient to ensure the recognition and enforcement of judgments abroad. Moreover, the existing legislative frameworks may be unable to capture and effectively regulate these misleading effects of forum marketing. The inadequacy of current legislation to effectively mitigate these potential effects of forum marketing suggests that, unlike ordinary courts, international commercial courts operate in ways that bring them closer to private corporate actors, where marketing practices are more common.

### B. Reconfiguring ‘Open Justice’

By marketing their procedural features, international commercial courts become more visible and break with the traditional perception of courts as anonymous and disinterested in public appearances. This is especially significant in civil law countries, which tend to emphasise the anonymity of the law and the collective, as opposed to the individual, reputation of the judiciary.^99^ Greater visibility is often associated with the public nature of court hearings and the right of the public to access court proceedings, referred to as the principle of ‘open justice’, which raises the question: does forum marketing increase court publicity by increasing the visibility of international commercial courts and their judges? Given that marketing targets specific audiences, an answer to this question would first require us to identify the target audiences of international commercial courts and their relevant external constituencies.

Depending on the different policy aims that animate their establishment, some international commercial courts reach out to a more domestic audience of litigants, while others reach out to a more international audience. Notwithstanding these differences, there is a common denominator between the different audiences of international commercial courts: the local bar. As discussed above in more detail, lawyers are the actual forum shoppers in international commercial disputes, and all international commercial courts are dependent on a domestic buy-in, especially during their early years of functioning. In turn, the expectation that international commercial courts would generate litigation business offered the bar a powerful incentive to participate in the corresponding rule making and to support the newly minted fora by offering them their maiden cases. The reciprocal relationships between international commercial courts and the local bar suggest that forum marketing reaches out to a specific audience, consisting primarily of domestic law firms that specialise in international commercial disputes. While lawyers have always been important to judges,^100^ they are especially important to international commercial court judges.

While forum marketing encourages the courts and their judges to reach out to prospective litigants, thereby targeting a broader audience, a niche of the legal profession, namely, the local bar that focuses on international commercial disputes, constitutes the most significant audience for international commercial courts and their judges. Thus, forum marketing in the context of international commercial courts does not increase the public’s access to court proceedings or judgments, but mainly contributes by helping the courts to raise awareness of their recent establishment with a view to acquiring a caseload. International commercial courts aim to attract international disputes and are therefore open to the world; yet, in the end, their world is small.

### C. The One Per Cent Procedure

By participating in the rule-making processes and steering cases towards the international commercial courts, lawyers could exert pressure on those courts to promote specific policies that they or their clients consider favourable.^101^ Subjecting the law and justice to market preferences empowers those social groups who are in a position to navigate the market. Bookman persuasively argues that forum selling in the context of international commercial courts may favour ‘repeat players’—namely, well-resourced parties who are experienced in litigation and have access to expert legal advice.^102^

While international commercial courts cater for cross-border commercial disputes, other types of disputes, such as domestic disputes, or cases involving family or criminal law, could be underestimated and undervalued.^103^ Cuts in legal aid,^104^ the removal of oral hearings for certain cases^105^ and the outsourcing of some cases to private dispute resolution providers suggest that the interest of national governments in improving the civil justice system is mainly focused on high-value, cross-border commercial claims, which are likely to end up before the international commercial courts. International commercial courts could therefore leave some courts, parties and lawyers behind, giving rise to a two-tier justice system and creating procedural inequality. Such concerns were indeed voiced in Belgium and explain the ultimate withdrawal from parliament of the proposal to create an international commercial court. The Brussels International Business Court prompted the characterisations ‘VIP’ and ‘caviar’ court.^106^ It was feared that the establishment of a specialised court for business-to-business disputes would give business litigants preferential treatment, create procedural inequality and thereby lead to a one per cent procedure.^107^ In addition, the claim that international commercial courts would generate income that could subsequently be poured into the ordinary courts and benefit the justice system as a whole is reminiscent of ‘trickle down’ economics, wherein tax cuts and benefits for the rich eventually trickle down and benefit the overall economy in the long term. However, the validity of these theories remains contested, and they are considered to contribute to income inequality.^108^ One lawyer who appeared before an international commercial court vividly described the special attention received by corporations and business litigants before international commercial courts: ‘Same people, same court in English. But it pays special attention because of its international character. If you go to a restaurant that you like and suddenly, they ask you to sit at the chef’s table, would you? Yes!’^109^

Forum marketing plays a crucial role in reinforcing this pro-business approach in civil justice. By labelling specific rules and procedures as ‘best practices’ and facilitating their dissemination, forum marketing has a persuasive and, by extension, normative force. It elevates and disseminates specific procedural rules or practices while downplaying others. Forum marketing encourages international commercial courts to resemble common law courts, in particular, the LCC, so that they appear more familiar and therefore more attractive to foreign business litigants and their lawyers. However, the use of English as the court language and the adoption of English rules of civil procedure do not only give away an attempt to appear familiar to foreign litigants. In the context of the international commercial courts established beyond Europe, their common law features additionally give away their attempts to overcome litigants’ distrust of foreign, non-Western legal systems. Theus draws a parallel between international commercial courts and the mixed courts of the colonial era, such as the Mixed Courts of Egypt, which were staffed by local and foreign judges and handled Egyptian–Western civil and commercial disputes in French.^110^ Mixed courts were considered a response to the inability of host states to abide by the ‘standard of civilisation’ and gain full membership of the international community.^111^ The link between international commercial courts and the mixed courts of the colonial era illustrates how the procedural features of international commercial courts could similarly reiterate and intensify biases towards Western superiority and exceptionalism. Forum marketing refreshes the memory structures of the colonial past and strategically employs the persuasive power of such biases. Consequently, forum marketing assumes away the regional and cultural contingencies of litigation and universalises rules and practices of a Western origin.

In addition to proceedings inspired by common law or arbitration, international commercial courts and their forum marketing prioritise party autonomy and efficiency. While party autonomy allows parties to design proceedings by mutual agreement and increases procedural flexibility, the heightened emphasis on party autonomy overlooks bargaining inequalities between contracting parties. Recent legislative developments that offer increased protection to small and medium-sized enterprises take into consideration the bargaining inequalities that can exist in commercial relationships and acknowledge that while commercial parties are, in principle, equal, some parties are more equal than others. In addition, the increased emphasis on party autonomy facilitates forum shopping, consequently favouring those jurisdictions that are in a position to attract international commercial disputes. This means that other jurisdictions lose their ability to decide these disputes, can no longer develop case law, and cease building judicial and legal expertise.^112^ In a similar vein, while economics could yield valuable insights for procedural reforms, the one-sided focus on procedural standards of a more economic nature, such as efficiency, could nevertheless overshadow other equally significant standards, for example, the right of both parties to be heard, even if this can result in delays and undermine efficiency.^113^ After all, the above analysis of international commercial courts’ common law features has illustrated that the courts do not necessarily prioritise the most efficient features, but the most familiar ones. Forum marketing thus reinforces a pro-business approach in civil procedure and private international law. Even if this prioritises party autonomy and efficiency, it has the negative effect of crowding out alternative normative standards that are more sensitive to legal culture, and are based on procedural justice, state sovereignty and the public responsibility of courts.^114^ Once entrenched, these procedural standards could easily find their way into ordinary court proceedings and have a broader spill-over effect on other procedural law reforms. The possibility of adopting some features of the NCC—such as its higher court fees—in ordinary court proceedings was fiercely debated during the parliamentary debate that preceded the adoption of the NCC law.^115^ More recently, in January 2022, a legislative decree extended the use of English as a court language to the ordinary Dubai courts.^116^

Lastly, by presenting specific procedural rules or practices as a direct response to parties’ preferences, forum marketing obscures the politics behind the creation of international commercial courts and the role of lawyers in their establishment.^117^ It creates the impression that international commercial courts are solely the product of a desire to improve national courts and justice systems. While international commercial courts could indeed improve international commercial dispute resolution, they were not established solely with this goal in mind, but also with the intention of attracting foreign investment and creating litigation business. By recasting public concerns as private, facilitated by the ‘technicality’ of private international law, forum marketing distracts from political considerations and reinforces the sometimes misplaced assumption that private law disputes and the related private international law matters are only significant to the parties in trial and therefore ‘value neutral’ or ‘apolitique’.^118^ However, the study of forum marketing in the context of international commercial courts reveals that private international law is a politically laden field of law, just like every other field of law.

The use of marketing practices in civil justice—traditionally considered as a space insulated from the market—reflects broader trends towards deregulation, liberalisation and privatisation characteristic of neoliberalism.^119^ The academic literature has considered how the law on one hand and the economics and politics of neoliberalism on the other intersect. It contends that the state and its legal institutions play a central role in creating the ground rules of neoliberalism and guaranteeing their enforcement.^120^ By focusing on different types of legal rules and their judicial interpretation, legal scholars have exposed the diverse ways in which the law facilitates capital accumulation and ultimately contributes to contemporary inequalities.^121^ There is less literature, however, on how civil procedure, and private international law more broadly, can play a part in creating wealth as well as inequality.^122^ The study of forum marketing reminds us of the redistributive effects of private international law and highlights that it is precisely the field’s technical nature that ironically presents itself as ideal for a neoliberal globalisation. Forum marketing fosters a pro-business approach that minimises the public role of justice at the expense of other disputes and parties.^123^ Hence, if one adopts a broader view of the role of courts as significant, not only to the parties in trial but also to third parties and society at large, forum marketing may be seen to have undesirable implications. Similar to other types of regulatory competition,^124^ forum marketing, even if not detrimental to the parties in trial, has negative externalities for the wider society.^125^ Unlike the literature on forum selling, according to which forum selling is only problematic in non-contractual disputes because it leads to pro-claimant approaches, this article argues that, in the context of international commercial courts, forum selling leads to a pro-business approach, irrespective of the nature of the underlying dispute, and that forum marketing plays a central role in reinforcing this approach and creating procedural inequality.

## 7. Conclusion

This article has identified that, unlike ordinary courts, international commercial courts go beyond forum selling and engage in forum marketing to attract cases. Forum marketing has largely escaped the academic literature, which presupposes that, just like the ordinary courts, international commercial courts are disinterested in public appearances and marketing. However, the increased competition in cross-border commercial dispute resolution is forcing international commercial courts to rise above the competition through marketing. Forum marketing comes with broader implications for civil justice. By encouraging courts and their judges to become more visible and attractive to litigants, it redefines the principle of ‘open justice’. It also prompts courts to adopt specific procedural features of a more marketing value and could therefore mislead litigants in their choice of court. More significantly, forum marketing is central to the promotion of a procedural model that is primarily designed with corporations and business litigants in mind. By exploiting memory and attention structures, forum marketing facilitates the dissemination and eventual prioritisation of procedural standards that are grounded in specific norms of a more Western and economic outlook. At the same time, forum marketing screens out the market forces that originally drove the establishment of international commercial courts, the power relations that underpin their functioning and the role of these courts in fostering a pro-business approach in civil justice. Forum marketing thus matters as much because of what it illuminates as what it obscures. It gives away a neoliberal ideology in civil justice which is premised upon a neocolonial and universalising mode of thinking, the priority of market values and the separation of law and politics.

According to the publicly available data, some international commercial courts have successfully attracted a significant caseload, while others have had a slower uptake.^126^ These differences in caseload could reflect varying degrees of popularity, but are also linked to the courts’ jurisdictional rules. Although some international commercial courts have a higher caseload than others, the number of opt-in cases, where both parties have chosen the court based on a choice-of-court agreement, is not high. The generally limited caseload of these international commercial courts could hint at their relative success and the limits of forum marketing. Stakeholders, however, emphasise that it takes time for courts to build a caseload, and international commercial courts hold a promising potential.^127^ Whether these statements reflect genuine optimism or marketing may be difficult to discern. In aid of such statements, marketing research underscores that successful marketing techniques require ‘a commitment over many years, even decades’. The impact of marketing on sales is spread out over time and is therefore difficult to measure.^128^ Returning to the iceberg metaphor, sales are only the tip of the marketing iceberg, but ‘we don’t know what proportion of the total iceberg the tip represents’.^129^ Future research shall inquire whether the challenge of international commercial courts to carve out their own piece of international commercial dispute resolution will abate over time, taking forum marketing with it. Alternatively, future studies could explore whether forum marketing will persist and expand to other courts and the judiciary. It shall also examine whether forum marketing extends even further to substantive law matters.

## Footnotes

1 Daniel Klerman, ‘Personal Jurisdiction and Product Liability’ (2012) 85 S Cal L Rev 1551, 1554; Daniel Klerman, ‘Rethinking Personal Jurisdiction’ (2014) 6 Journal of Legal Analysis 245, 259; Daniel Klerman, ‘Forum Selling and Domain-Name Disputes’ (2016) 48 Loy U Chi LJ 561; Daniel Klerman and Greg Reilly, ‘Forum Selling’ (2016) 89 S Cal L Rev 241; Stefan Bechtold, Jens Frankenreiter and Daniel Klerman, ‘Forum Selling Abroad’ (2019) 92 S Cal L Rev 487.

2 Jonas Anderson, ‘Court Competition for Patent Cases’ (2015) 163 U Pa L Rev 631, 668–77; Klerman and Reilly (n 1) 250–70; Bechtold, Frankenreiter and Klerman (n 1) 502–13, 523–34.

3 Lynn LoPucki, *Courting Failure: How Competition for Big Cases Is Corrupting the Bankruptcy Courts* (University of Michigan Press 2005) 241; Anderson (n 2) 679; Klerman and Reilly (n 1) 245; Bechtold, Frankenreiter and Klerman (n 1) 512–53, 526–31.

4 Xandra Kramer and John Sorabji (eds), *International Business Courts: A European and Global Perspective* (Eleven International Publishing 2019); Pamela Bookman, ‘The Adjudication Business’ (2020) 45 Yale J Int’l L 227; Matthew Erie, ‘The New Legal Hubs: The Emergent Landscape of International Commercial Dispute Resolution’ (2020) 60 Va J Int’l L 225; Stavros Brekoulakis and Georgios Dimitropoulos (eds), *International Commercial Courts: The Future of Transnational Adjudication* (CUP 2022).

5 eg Geert van Calster, ‘Brexit and the Competition of Dispute Resolution Fora in Europe’ in Brekoulakis and Dimitropoulos (n 4) 501.

6 Bookman (n 4) 264. See also Burkhard Hess and Timon Boerner, ‘Chambers for International Commercial Disputes in Germany: The State of Affairs’ (2019) 12 Erasmus Law Review 33.

7 Klerman, ‘Personal Jurisdiction’ (n 1) 1554; Klerman, ‘Rethinking’ (n 1) 259; Klerman, ‘Forum Selling and Domain-Name Disputes’ (n 1) 561; Klerman and Reilly (n 1) 241; Bechtold, Frankenreiter and Klerman (n 1) 487.

8 Anderson (n 2) 679, 668–77; Klerman and Reilly (n 1) 245, 250–70; Bechtold, Frankenreiter and Klerman (n 1) 502–13, 523–34.

9 Pamela Bookman, ‘Arbitral Courts’ (2021) 61 Va J Int’l L 161; Thomas Schultz and Clément Bachmann, ‘International Commercial Courts: Possible Problematic Social Externalities of a Dispute Resolution Product with Good Market Potential’ in Brekoulakis and Dimitropoulos (n 4) 52; Man Yip, ‘The Battle for Jurisdiction through Jurisdictional Requirements: Comparing the Commercial Court of England and Wales, the Singapore International Commercial Court and the Chinese International Commercial Court’ in Brekoulakis and Dimitropoulos (n 4) 176; Georgia Antonopoulou, ‘The “Arbitralization” of Courts: The Role of International Commercial Arbitration in the Establishment and the Procedural Design of International Commercial Courts’ (2023) 14 JIDS 328.

10 Michael J Baker, ‘What Is Marketing?’ in Michael J Baker and Susan Hart (eds), *The Marketing Book* (Taylor & Francis 2016) 7.

11 Gary Armstrong and Philip Kotler, *Marketing: An Introduction* (14th edn, Pearson 2020) 58. See also Theodore Levitt, ‘Marketing Myopia’ (2004) 82 Harvard Business Review 138.

12 ibid 31.

13 Byron Sharp, *How Brands Grow: What Marketers Don’t Know* (OUP 2010) 28–38; Baker (n 10).

14 Edmund Jerome McCarthy, *Basic Marketing: A Managerial Approach* (Homewood III 1960). See also Neil H Borden, ‘The Concept of the Marketing Mix’ (1984) 2 Journal of Advertising Research 7.

15 Philip Kotler and Sidney J Levy, ‘Broadening the Concept of Marketing’ (1969) 33 Journal of Marketing 10. See also Philip Kotler, ‘A Generic Concept of Marketing’ (1972) 36 Journal of Marketing 46.

16 Kotler and Levy (n 15).

17 Baker (n 10) 4–5; Armstrong and Kotler (n 11) 54.

18 Yves Dezalay and Bryant Garth, *Dealing in Virtue, International Commercial Arbitration and the Construction of a Transnational Legal Order* (The University of Chicago Press 1996) 34–41; Chistopher R Drahozal, ‘Contracting Out of National Law: An Empirical Look at the New Law Merchant’ (2005) 80 Notre Dame L Rev 523; Ralf Michaels, ‘Dreaming Law Without a State: Scholarship on Autonomous International Arbitration as Utopian Literature’ (2013) 1 Lond Rev Int Law 37.

19 Christine Schwöbel-Patel, *Marketing Global Justice: The Political Economy of International Criminal Law* (CUP 2021).

20 Leslie J Moran, ‘Managing the “Critical Independencies” of the Media and Judiciary in the United Kingdom: A Study of the Emergence of “Judicial Communications” Media Initiatives’ in Micheal Asimow and others (eds), *Law and Popular Culture: International Perspectives* (Cambridge Scholars Publishing 2014) 195; Leslie J Moran, ‘Mass-Mediated Open Justice: Court and Judicial Reports in the Press in England and Wales’ (2014) 34 LS 143.

21 Georgia Antonopoulou and Xandra Kramer, ‘A New Court Open for International Business Soon: The Commercial Court in Cyprus’ (*ConflictsofLaws.net*, 26 February 2023) <https://conflictoflaws.net/2023/a-new-court-open-for-international-business-soon-the-commercial-court-in-cyprus/> accessed 20 March 2024; Law to Strengthen Germany as a Place for Litigation through the Establishment of Commercial Courts and the Introduction of English as the Court Language in Civil Courts (*Gesetz zur Stärkung des Justizstandortes Deutschland durch Einführung von Commercial Courts und der Gerichtssprache Englisch in der Zivilgerichtsbarkeit*) (Ministry of Justice, 13 August 2023)<www.bmj.de/SharedDocs/Gesetzgebungsverfahren/DE/2023_Commercial_Courts_Justiz_Staerkungsgesetz.html?nn=110490> accessed 20 March 2024; ‘Singapore and Bahrain Sign Bilateral Treaty on Appeals from the Bahrain International Commercial Court’ (Ministry of Law, Singapore, 20 March 2024).

22 Anderson (n 2) 631, 664–5; Klerman and Reilly (n 1) 272–5; Bechtold, Frankenreiter and Klerman (n 1) 519–20.

23 World Bank Group, *Doing Business 2020: Comparing Business Regulation in 190 Economies* (2020) 91, 96, 98, 109, 126; World Bank Group, *Business Enabling Environment* (2022).

24 Raad voor de Rechtspraak (Council for the Judiciary), ‘Plan tot oprichting van de Netherlands Commercial Court’ (‘Plan for the Establishment of the Netherlands Commercial Court’) (November 2015) 7 <www.rechtspraak.nl/SiteCollectionDocuments/plan-netherlands-commercial-court.pdf> (NCC Plan); ‘Qatar International Court Contributes in Two of the World Bank’s Indexes’ (QICDRC, 27 October 2019) <https://thepeninsulaqatar.com/article/27/10/2019/Qatar-International-Court-contributes-to-enhancing-World-Bank’s-Qatar-Indexes> both accessed 20 March 2024.

25 Bookman (n 4) 240.

26 Lord Woolf in C Campbell-Holt (ed), *A Vision of the AIFC Court* (AIFC Court 2019) 8.

27 NCC Plan (n 24) 7. See also Deutscher Bundestag, ‘Entwurf eines Gesetzes zur Stärkung der Gerichte in Wirtschaftsstreitigkeiten’ (‘Legislative Proposal for the Strengthening of Courts in Commercial Disputes’) (27 April 2022) Explanatory Statement, 11 (German Legislative Proposal).

28 Singapore International Commercial Court Committee, ‘Report of the Singapore International Commercial Court Committee’ (2013) §4(a) (SICC Committee Report).

29 Indranee Rajah, ‘Speech’ (Litigation Conference, 16 March 2015) §15, 25 <www.mlaw.gov.sg/news/speeches/speech-by-senior-minister-of-state-for-law--indranee-rajah--at-t/> accessed 20 March 2024.

30 NCC Plan (n 24) 8.

31 Anderson (n 2) 661–6; Klerman and Reilly (n 1) 272; Bechtold, Frankenreiter and Klerman (n 1) 513, 535–6.

32 Wet griffierechten in burgerlijke zaken (Act on Court Fees in Civil Proceedings), art 9a.

33 For a comparison, see Xandra Kramer and Georgia Antonopoulou, ‘Commercializing Litigation: The Case of the Netherlands Commercial Court’ in Albert Henke and others (eds), *International Commercial Courts. A Paradigm for the Future of Adjudication?* (Edizioni Scientifiche Italiane 2023) 105, 115.

34 SICC Practice Directions, Part VII, Fees.

35 German Legislative Proposal, arts 4 and 5.

36 Boston Consulting Group, ‘Marktverkenning Netherlands Commercial Court’ (‘Market Survey Netherlands Commercial Court’) 6 (Market Survey NCC).

37 Kamerstukken II 2017/2018 (Parliamentary Papers II), 34761, B, Wijziging van het Wetboek van Burgerlijke Rechtsvordering en de Wet Griffierechten Burgerlijke Zaken in Verband Met Het Mogelijk Maken van Engelstalige Rechtspraak bij de International Handelskamers van de Rechtbank Amsterdam en het Gerechtshof Amsterdam (Amendments to the Code of Civil Procedure and the Civil Court Fees Act with Regard to the Introduction of English-language Case Law at the International Commercial Chambers of the Amsterdam District Court and the Amsterdam Court of Appeal), Memorie van Antwoord (Statement of Reply) (31 May 2018) 5.

38 Anderson (n 2) 661–2; Klerman and Reilly (n 1) 271; Bechtold, Frankenreiter and Klerman (n 1) 514, 535. See also Lawrence Baum, *Judges and Their Audiences: A Perspective on Judicial Behavior* (Princeton UP 2006) 153, 205.

39 Klerman and Reilly (n 1) 275–7.

40 ‘Judges’ (DIFC Courts) <Judges | DIFC Courts>; ‘The Court' (QICDRC) <The Court | QICDRC>; ‘Judges’ (ADGM Courts) <www.adgm.com/adgm-courts/judges>; ‘Justices’ (AIFC Court) <https://court.aifc.kz/who-we-are/justices/>; ‘Judges’ (SICC) <www.sicc.gov.sg/about-the-sicc/judges> all accessed 20 March 2024.

41 See Eddy Bauw, ‘Commercial Litigation in Europe in Transformation: The Case of the Netherlands Commercial Court’ (2019) 12 Erasmus Law Review 17.

42 See also Alyssa King and Pamela Bookman, ‘Traveling Judges’ (2022) 116 AJIL 502.

43 SICC Rules, Ord 2, r 1; Protocol on Procedural Rules Applicable to the International Chamber of the Paris Commercial Court (21 February 2018), art 1.3 (Protocol Paris International Chamber); ‘Protocol on Procedural Rules Applicable to the International Chamber of the Court of Appeal of Paris’ (Avocat Paris, 2018), art 1.1 <www.cours-appel.justice.fr/sites/default/files/2019-04/Traduction%20en%20anglais%20du%20protocole%20CCIP-CA%20-%20V4%20.pdf> (Protocol Paris International Chamber of the Court of Appeal); ‘Chamber for International Commercial Disputes’ (Ordentliche Gerichtsbarkeit Hessen) <https://ordentliche-gerichtsbarkeit.hessen.de/landgerichtsbezirk-frankfurt-am-main/landgericht-frankfurt-am-main/kammer-fuer-internationale-handelssachen>; Wetboek van Burgerlijke Rechtsvordering (Dutch Code of Civil Procedure), art 30r(1) (DCCP); Council for the Judiciary, ‘Rules of Procedure for the International Commercial Chambers of the Amsterdam District Court (NCC District Court) and the Amsterdam Court of Appeal (NCC Court of Appeal)’ (3rd edn, June 2023) art 1.3.1(c); Supreme People’s Court, ‘Provisions on Several Issues Regarding the Establishment of the International Commercial Court’ (CICC Provisions) art 2(1).

44 Interview with court personnel (6 June 2019).

45 Interview with court personnel (18 February 2020).

46 Stefan Vogenauer, ‘Regulatory Competition through Choice of Contract Law and Choice of Forum in Europe: Theory and Evidence’ (2013) 21 European Review of Private Law 53; Erlis Themeli, ‘Matchmaking International Commercial Courts and Lawyers’ Preferences in Europe’ (2019) 12 Erasmus Law Review 75.

47 Stefan Vogenauer, ‘Perceptions of Civil Justice Systems in Europe and Their Implications for Choice of Forum and Choice of Contract Law: An Empirical Analysis’ in Stefan Vogenauer and Chris Hodges (eds), *Civil Justice Systems in Europe: Implications for Choice of Forum and Choice of Contract Law* (Hart Publishing 2009) Question 33; Themeli, ‘Matchmaking’ (n 46) 75.

48 Gillian K Hadfield, ‘The Price of Law: How the Market for Lawyers Distorts the Justice System’ (1999–2000) 98 Mich L Rev 968; Gary Low, ‘A Psychology of Choice of Laws’ (2013) 24 European Business Law Review 372; Vogenauer, ‘Regulatory Competition’ (n 45) 19–22.

49 Vogenauer, ‘Regulatory Competition’ (n 46) 42, 43; Erlis Themeli, *The Great Race of Courts: Civil Justice System Competition in the European Union* (Eleven International Publishing 2018) 299.

50 See White & Case and School of International Arbitration, Queen Mary University of London, ‘2010 International Arbitration Survey: Choices in International Arbitration’, Chart 7; Themeli, *The Great Race of Courts* (n 49) 174–82.

51 Hadfield (n 48) 971–2.

52 Market Survey NCC (n 36) 2, 8, 10, 16; NCC Plan (n 24) 9.

53 See ‘News’ (NCC) <www.rechtspraak.nl/English/NCC/news/Pages/default.aspx>; ‘Executive Team’ (DIFC Courts) <www.difccourts.ae/about/court-structure/executive-team> accessed 20 March 2024.

54 DIFC Courts, *Annual Report* (2022) 37 <www.difccourts.ae/media-centre/publications/annual-reports/annual-report-2022-english> accessed 20 March 2024.

55 See SICC, *Annual Report* (2022) <www.judiciary.gov.sg/AnnualReport2022/SP-Developments-Singapore-Court.html> accessed 20 March 2024.

56 ‘Who We Are’ (AIFC Court) <https://court.aifc.kz/en/who-we-are> accessed 20 March 2024.

57 Interview with court personnel (6 June 2019).

58 David Arnold, *The Handbook of Brand Management* (Century Business 1992); Stuart Crainer, *The Real Power of Brands: Making Brands Work for Competitive Advantage* (FT Pitman Publishing 1995).

59 At the time of writing, these international commercial courts do not protect their brand names through intellectual property law and therefore the use of brand names lacks legal implications. For the International Criminal Court, which has registered its logo as a trademark, see Schwöbel-Patel (n 19) 42–3.

60 On the significance of distinctiveness in branding, see Sharp (n 13) 112.

61 DCCP (n 43) art 30r; NCC Rules, art 1.1.1.

62 Constitution of the Republic of Singapore, art 94(1)(b)(ii); Supreme Court of Judicature Act, s 18A.

63 Man Yip, ‘The Singapore International Commercial Court: The Future of Litigation?’ (2019) 12 Erasmus Law Review 82.

64 Explanatory Statement NCC Law, 5–6; German Legislative Proposal, Explanatory Statement, 14; Emmanuel Jeuland, ‘The International Chambers of Paris: A Gaul Village’ in Kramer and Sorabji (n 4) 69–70.

65 Georgia Antonopoulou, ‘Requirements upon Agreements in Favour of the NCC and the German Chambers—Clashing with the Brussels Ibis Regulation?’ (2019) 12 Erasmus Law Review 56; Pietro Ortolani and Bas van Zelst, ‘International Commercial Courts and EU Law: Easing the Tension’ (2023) 14 JIDS 76.

66 Lars Bergkvist and Charles R Taylor, ‘Leveraged Marketing Communications: A Framework for Explaining the Effects of Secondary Brand Associations’ (2016) 6 AMS Review 157.

67 ‘NCC’ (Rechtspraak) <www.rechtspraak.nl/english/ncc/Pages/default.aspx> accessed 20 March 2024.

68 Yu Chen, ‘The First Anniversary of China International Commercial Courts’ (China Justice Observer, 18 August 2019) <www.chinajusticeobserver.com/a/review-the-first-anniversary-of-china-international-commercial-courts> accessed 20 March 2024.

69 Yong Gan, ‘Jurisdiction Agreements in Chinese Conflict of Laws: Searching for Ways to Implement the Hague Convention on Choice of Court Agreements in China’ (2018) 14 J Priv Int’l L 304.

70 See n 40.

71 King and Bookman (n 42) 42.

72 ‘The Supreme People’s Court Established the International Commercial Expert Committee’ (CICC) <http://cicc.court.gov.cn/html/1/219/208/209/981.html> accessed 20 March 2024.

73 ‘Expert Committee’ (CICC) <http://cicc.court.gov.cn/html/1/219/235/237/index.html> accessed 20 March 2024.

74 Dezalay and Garth (n 18) 34–41.

75 Legal High Committee for Financial Markets of Paris, ‘Recommendations for the Creation of Special Tribunals for International Business Disputes’ (3 May 2017) §21 (Committee for Financial Markets of Paris, Recommendations); interview with court personnel (18 February 2020).

76 Jayanth K Krishnan, *The Story of The Dubai International Financial Center Courts: A Retrospective* (DIFC Academy of Law 2018) 10.

77 SICC Committee Report (n 28) §2.

78 Market Survey NCC (n 36) figs 4 and 11.

79 Interview with lawyer (16 May 2019).

80 ibid.

81 Civil Procedure Law, arts 11 and 262.

82 Market Survey NCC (n 36) 4; Committee for Financial Markets of Paris, Recommendations, §21; German Legislative Proposal, Explanatory Statement, 5.

83 See Gerichtsverfassungsgesetz (Courts Constitution Act) (9 May 1975), art 185(2).

84 Protocol Paris International Chamber, arts 2.2–2.6 and 7; Protocol Paris International Chamber of the Court of Appeal, arts 2.1–2.4, 3.3 and 4.4.2.

85 Bundesrechtsanwaltskammer (German Federal Bar Association), ‘Stellungnahme Nr 23/2014 Juni 2014 zum Entwurf eines Gesetzes zur Einführung von Kammern für internationale Handelssachen’ (‘Statement Nr 23/2014 June 2014 with regard to the Legislative Proposal for the Establishment of Chambers for International Commercial Disputes’); Market Survey NCC (n 36) fig 2.

86 Schultz and Bachmann (n 9) 56; Antonopoulou (n 9) 2.

87 ‘Protocols and Memoranda’ (DIFC Courts) <www.difccourts.ae/about/protocols-memoranda?ccm_paging_p=2&ccm_order_by=ak_date&ccm_order_by_direction=desc>. See also Jayanth Krishnan and Priya Purohit, ‘A Common Law Court in an Uncommon Environment: The DIFC Judiciary and Global Commercial Dispute Resolution’ (2014) 25 American Review of International Arbitration 497, 512.

88 Nandia Alexander and others, ‘International Dispute Resolution Survey: Currents of Change, Singapore International Dispute Resolution Academy’ (Singapore Management University 2021) §8.1.5.

89 Convention on the Recognition and Enforcement of Foreign Arbitral Awards (adopted 10 June 1958, entered into force 7 June 1959).

90 Hague Convention on Choice of Court Agreements (30 June 2005).

91 Hague Convention on the Recognition and Enforcement of Foreign Judgments in Civil or Commercial Matters (2 July 2019).

92 Krishnan and Purohit (n 87) 510–12; Krishnan (n 76) 59.

93 DIFC Courts, ‘Strategic Plan’, 28.

94 ‘Enforcement of Money Judgements’ (SICC) <www.judiciary.gov.sg/singapore-international-c>.

95 Interview with court personnel (18 February 2020).

96 See SICC Committee Report (n 28) §51; Gilles Cuniberti, ‘Signaling the Enforceability of the Forum’s Judgments Abroad’ [2020] Rivista di Diritto Internazionale Privato e Prozessuale 33.

97 Cuniberti (n 96) 52.

98 ibid 52–4.

99 See also Nuno Garupa and Tom Ginsburg, *Judicial Reputation: A Comparative Theory* (The University of Chicago Press 2015) 9–10.

100 Baum (n 38) 73.

101 LoPucki (n 3) 19–24. See also Jonathan R Macey and Geoffrey P Miller, ‘Toward an Interest-Group Theory of Delaware Corporate Law’ (1987) 65 Tex L Rev 472; Lawrence Baum, ‘Probing the Effects of Judicial Specialization’ (2009) 58 Duke LJ 1678; Ralf Michaels, ‘Make or Buy—A Public Market for Legal Transplants?’ in Horst Eidenmüller (ed), *Regulatory Competition in Contract Law and Dispute Resolution* (Beck-Hart-Nomos 2013) 28.

102 Bookman (n 4) 267. See also Schultz and Bachmann (n 9).

103 Brooke D Coleman, ‘One Percent Procedure’ (2016) 91 Wash L Rev 1005, 1041; Alyssa King, ‘Global Civil Procedure’ (2021) 62 Harv Int’l LJ 252.

104 In England, see Legal Aid, Sentencing and Punishment of Offenders Act 2012.

105 In England, see Practice Direction 51ZC—The Small Claims Paper Determination Pilot <www.justice.gov.uk/courts/procedure-rules/civil/rules/part51/practice-direction-51zc-the-small-claims-paper-determination-pilot> accessed 20 March 2024. For a discussion, see Kate Leader, ‘The Small Claims Paper Determination Pilot: Filtering Out the County Courts’ “Garbage Claims”’ (*Modern Law Review Forum*, 18 May 2022) <www.modernlawreview.co.uk/leader-small-claims-pilot/> accessed 20 March 2024.

106 Geert van Calster, ‘The Brussels International Business Court: A Carrot Sunk by Caviar’ in Kramer and Sorabji (n 4) 107.

107 Coleman (n 103).

108 See Joseph Stiglitz, ‘Inequality and Economic Growth’ (2015) 86 Political Quarterly 134; David Hope and Julian Limberg, ‘The Economic Consequences of Major Tax Cuts for the Rich’ (January 2020) Working Paper LSE 55.

109 Interview with lawyer (3 October 2019).

110 Willem Theus, ‘There and Back Again: From Consular Courts through Mixed Arbitral Tribunals to International Commercial Courts’ in Hélène Ruiz Fabri and Michel Erpelding (eds), *The Mixed Arbitral Tribunals, 1919–1939: An Experiment in the International Adjudication of Private Rights* (Nomos 2023) 45, 54.

111 ibid 36, 46; Michel Erpelding, ‘Mixed Courts of the Colonial Era’ in Hélène Ruiz Fabri (ed), *Max Planck Encyclopedia of International Procedural Law* (OUP 2020).

112 Lucas Clover Alcolea, ‘The Rise of the International Commercial Court: A Threat to the Rule of Law?’ (2022) 13 JIDS 413, 424–32.

113 Ralf Michaels, ‘Comparative Law by Numbers? Legal Origins Thesis, Doing Business Reports, and the Silence of Traditional Comparative Law’ (2009) 57 Am J Comp L 773.

114 See also Alex Mills, ‘The Privatisation of Private (and) International Law’ (2023) 76 CLP 75.

115 Parliamentary Papers I 2017/18 (Kamerstukken I), 34 761 C, Amendments to the Code of Civil Procedure and the Civil Court Fees Act with regard to the introduction of English-language case law at the international commercial chambers of the Amsterdam District Court and the Amsterdam Court of Appeal (Wijziging van het Wetboek van Burgerlijke Rechtsvordering en de Wet griffierechten burgerlijke zaken in verband met het mogelijk maken van Engelstalige rechtspraak bij de internationale handelskamers van de rechtbank Amsterdam en het gerechtshof Amsterdam) 18 June 2018.

116 Federal Decree Law No 42 of 2022 (The New Civil Procedures Law).

117 King (n 103) 282.

118 See Symeon Symeonides, *Codifying Choice of Law Around the World: An International Comparative Analysis* (OUP 2014) 344.

119 See also Schwöbel-Patel (n 19) 26.

120 Honor Brabazon (ed), *Neoliberal Legality: Understanding the Role of Law in the Neoliberal Project* (Routledge 2017); Quinn Slobodian, *Globalists: The End of Empire and the Birth of Neo-Liberalism* (Harvard UP 2018).

121 Katharina Pistor, *The Code of Capital* (Princeton UP 2019); Eric Posner and Cass Sunstein, ‘Antitrsut and Inequality’ (2022) 2 American Journal of Law and Equality 190; Joseph Spooner, ‘Contract Law When the Poor Pay More’ (2024) 20 OJLS 1. See also Antoine Vauchez and Pierre France, *The Neoliberal Republic: Corporate Lawyers, Statecraft, and the Making of Public-Private France* (Cornell UP 2020).

122 Marc Galanter, ‘Why the “Haves” Come Out Ahead: Speculations on the Limits of Legal Change’ (1974) 9 L & Soc’y Rev 95. See also Coleman (n 103) 1041.

123 See also Curtis J Milhaupt and Katharina Pistor, *Law & Capitalism: What Corporate Crises Reveal About Legal Systems and Economic Development Around the World* (The University of Chicago Press 2008) 213; Michael J Sandel, *What Money Can’t Buy, The Moral Limits of Markets* (Penguin 2013); Schwöbel-Patel (n 19) 269.

124 See Lucian Arye Bebchuk, ‘Federalism and the Corporation: The Desirable Limits on State Competition in Corporate Law’ (1992) 105 Harv L Rev 1435.

125 Schultz and Bachmann (n 9) 52.

126 For the caseload of the NCC, see ‘NCC’ (Rechtspraak) <www.rechtspraak.nl/English/NCC/Pages/judgments.aspx>; for the Stuttgart Commercial Court, see ‘Successful Conflict Resolution at the 31st Chamber of Commercial Affairs’ (Commercial Court Stuttgart-Mannheim, 17 May 2022) <www.commercial-court.de/en/press-news/detail?tx_news_pi1%5Baction%5D=detail&tx_news_pi1%5Bcontroller%5D=News&tx_news_pi1%5Bnews%5D=19&cHash=2ba758bd8f4df8eec94f31864cd2a87b>; for the SICC, see ‘SICC’ (Singapore Courts) <www.sicc.gov.sg/hearings-judgments/judgments>; for the DIFC Courts, see DIFC Courts, *Annual Report* (2022) 23 <www.difccourts.ae/media-centre/publications/annual-reports/annual-report-2022-english>; for the QIC, see ‘Judgments’ (QICDRC) <www.qicdrc.gov.qa/judgments>; for the ADGM Courts, see ‘Cases’ (ADGM Courts) <www.adgm.com/adgm-courts/cases>; for the AIFC Court, see ‘More than 2,100 Cases Resolved and Enforced by the AIFC Court and IAC’ (AIFC Court, 7 June 2023) <https://court.aifc.kz/en/news/more-than-2-100-cases-resolved-and-enforced-by-the-aifc-court-and-iac>; for the CICC, see US–China Economic and Security Review Commission, *Dispute Settlement with Chinese Characteristics: Assessing China’s International Commercial Court* (28 February 2023) all accessed 20 March 2024.

127 See ‘The Netherlands Commercial Court: Settling International Business Disputes in English’ (*I amsterdam*, 5 March 2024) <www.iamsterdam.com/en/business/the-netherlands-commercial-court-settling-business-disputes-in-english> accessed 20 March 2024.

128 Sharp (n 13) 131, 137.

129 ibid 139.

